# Youth Ending the HIV Epidemic (YEHE): Protocol for a pilot of an automated directly observed therapy intervention with conditional economic incentives among young adults with HIV

**DOI:** 10.1371/journal.pone.0289919

**Published:** 2023-12-22

**Authors:** Marie C. D. Stoner, Kristin Ming, Danielle Wagner, Louis Smith, Henna Patani, Adam Sukhija-Cohen, Mallory O. Johnson, Sue Napierala, Torsten B. Neilands, Parya Saberi

**Affiliations:** 1 Women’s Global Health Imperative, RTI International, Berkeley, California, United States of America; 2 Center for AIDS Prevention Studies, University of California San Francisco, San Francisco, California, United States of America; 3 AIDS Healthcare Foundation, Los Angeles, California, United States of America; University of Manitoba Faculty of Health Sciences, NIGERIA

## Abstract

**Background:**

Young adults have a disproportionately high rate of HIV infection, high rates of attrition at all stages of the HIV care continuum, and an elevated probability of disease progression and transmission. Tracking and monitoring objective measures of antiretroviral therapy (ART) adherence in real time is critical to bolster the accuracy of research data, support adherence, and improve clinical outcomes. However, adherence monitoring often relies on self-reported and retrospective data or requires additional effort from providers to understand individual adherence patterns. In this study, we will monitor medication-taking using a real-time objective measure of adherence that does not rely on self-report or healthcare providers for measurement.

**Methods:**

The Youth Ending the HIV Epidemic (YEHE) study will pilot a novel automated directly observed therapy-conditional economic incentive (aDOT-CEI) intervention to improve ART adherence among youth with HIV (YWH) in California and Florida who have an unsuppressed HIV viral load. The aDOT app uses facial recognition to record adherence each day, and then economic incentives are given based on a participant’s confirmed adherence. We will enroll participants in a 3-month pilot study to assess the feasibility and acceptability of the aDOT-CEI intervention using predefined metrics. During and after the trial, a subsample of the pilot participants and staff/providers from participating AIDS Healthcare Foundation (AHF) clinics will participate in individual in-depth interviews to explore intervention and implementation facilitators and barriers.

**Discussion:**

YEHE will provide data on the use of an aDOT-CEI intervention to improve adherence among YWH who are not virologically suppressed. The YEHE study will document the feasibility and acceptability and will explore preliminary data to inform a trial to test the efficacy of aDOT-CEI. This intervention has the potential to effectively improve ART adherence and virologic suppression among a key population experiencing health disparities.

**Trial registration:**

The trial registration number is NCT05789875.

## Introduction

Young adults have a disproportionately high rate of HIV infection, high rates of attrition at all stages of the HIV care continuum, and a high probability of disease progression and transmission [1, 2]. Despite improvements in life expectancy among people with HIV and reductions in HIV transmission with increased availability of antiretroviral therapy (ART), the effectiveness of ART depends on high levels of adherence. In the United States (US), 59% of young adults with HIV who initiate ART are not retained in care and 63% achieve viral suppression [3]. These figures are dramatically lower than those for older adults [2, 4–6]. In addition to increased onward HIV transmission, poor adherence can increase drug resistance, leading to less effective treatment options and worse clinical outcomes [7]. Consequently, youth with HIV (YWH) represent a priority population in which to address low ART adherence.

Tracking and monitoring objective measures of ART adherence in real time is critical for strategies to support adherence and improve clinical outcomes. However, adherence monitoring often relies on self-reported retrospective data or requires additional effort from providers to understand individual adherence patterns, making it difficult for providers to accurately determine how to support their patients in real time. Young adults are the largest group of consumers of technology and internet use; over 96% of individuals ages 18–29 in the US have a smartphone [8–11]. Growing up in a technology-dominated era, young adults may be more comfortable with technology-mediated forms of communication than with face-to-face interactions. Therefore, technology-based strategies may be a good solution for monitoring adherence in YWH.

Technology-based directly observed therapy (DOT) strategies have been shown to be more cost-effective [12] and reduce provider and patient burden [13] compared with regular monitoring visits for medication adherence. While some evidence suggests that these technologies are feasible, acceptable, and effective for DOT, limited evidence exists describing digital DOT interventions in the context of HIV prevention and treatment. Our team conducted a scoping review to understand the current evidence related to the acceptability, feasibility, and efficacy of digital health technologies for DOT to monitor medication adherence in any disease state and included quantitative or qualitative studies published until January 8, 2020 [14]. All 28 identified studies found digital DOT to be acceptable and feasible and noted that digital DOT provided more autonomy and reduced cost and time for patients and providers compared with in-person DOT. Several studies showed increased medication adherence for treatment of tuberculosis [15], stroke [16], and schizophrenia [17]. Of the 28 studies, only one used digital DOT with an HIV prevention outcome [18]. Among men who have sex with men in San Francisco, automated DOT (aDOT), a technology-based DOT approach using artificial intelligence, was highly acceptable; 84% reported the smartphone application (app) helped with taking pre-exposure prophylaxis (PrEP), and median PrEP adherence was high (91%) [18].

Research also shows that monetary incentives within digital mental health interventions increase the regularity and volume of app use, and this increase in consistent use can improve health outcomes [19]. A meta-analysis of reinforcement interventions found that conditional economic incentives (CEI) significantly improved medication adherence for all health conditions evaluated relative to control conditions [20]. Other studies among people with HIV in the US have shown that financial incentives increased ART adherence [21–24] and HIV viral suppression [21, 22, 25, 26]. Studies used case managers, providers [21, 25], or Medication Event Monitoring System (MEMS) pill bottle caps to monitor adherence [22–24]. Digital technology with real-time information on adherence may provide an easier method to monitor and reward adherence in closer proximity to the behavior, which may have a strong effect on behavior change [26]. CEIs increase the perceived benefit of adherence, increase benefits and satisfaction related to adherence, and provide immediate positive rewards for adhering to ART, thus promoting behavior change [27]. In our study, we will take advantage of the combination of aDOT and CEIs to influence ART adherence among YWH.

### Study objectives

The Youth Ending the HIV Epidemic (YEHE) study will pilot an aDOT-CEI (automated directly observed therapy + conditional economic incentives) intervention, informed by the operant framework of Key Principles in Contingency Management Implementation [28], to improve ART adherence among YWH (18–29) in California and Florida who have an unsuppressed HIV viral load. AiCure, the aDOT platform that will be used in this study, is an evidence-based app that has been shown to improve adherence to medications for several health conditions, including PrEP in young men who have sex with men [18], hepatitis C treatment [29], schizophrenia [17], and stroke [16]. Our hypothesis is that the aDOT-CEI intervention to address ART adherence among YWH who are not virologically suppressed will have high feasibility and acceptability. Our research team includes a multidisciplinary group of researchers from the University of California, San Francisco (UCSF) and Research Triangle Institute (RTI) International, a community partnership with the nonprofit AIDS Healthcare Foundation (AHF), community input from a Youth Advisory Panel (YAP), and the Artificial Intelligence company AiCure.

## Materials and methods

### Overview of study design

YEHE aims to assess the feasibility and acceptability of a novel aDOT-CEI intervention. It will provide quantitative and qualitative evidence on feasibly and acceptability based on *a priori* criteria to determine whether a future trial is merited. We will conduct a single-arm pilot study among 30 YWH and collect survey data after 3 months of app use. After the trial, we will explore intervention and implementation facilitators/barriers by conducting in-depth qualitative interviews with 15 YWH and 5 staff/providers from participating AHF clinics.

### Study setting

The study will include YWH in Ending the HIV Epidemic counties in California and in Florida. In 2019, YWH accounted for 37% and 29% of all new HIV diagnoses in California and Florida, respectively [30]. California data also show that YWH had the lowest retention in care (52–54%) and virologic suppression (62–66%) as compared with other age groups [30]. Youth will be patients at AHF clinics in California and Florida. All research activities will be conducted remotely, thus obviating the need to travel for in-person study visits.

### Study population and Inclusion criteria

The pilot study will include (1) YWH ages 18–29-year-old (2) who have access to a smartphone, (3) speak/read English, (4) are clients at an AHF clinic in California and Florida, (5) consent to participate in the study, (6) are at least 3 months post HIV diagnosis and have an unsuppressed HIV viral load as determined by AHF clinical records and confirmed by participant, and (7) are taking a once-daily ART regimen. We will exclude anyone taking a twice daily regimen or anyone on long-acting injectable ART. YWH with a new HIV diagnosis in the past 3 months will be excluded.

### Youth Advisory Panel (YAP)

To inform study implementation, we will invite YWH from AHF sites in California and Florida to form the study YAP and seek their input throughout the study on the financial incentive structure, study recruitment and retention strategies, configuration of the AiCure app, and dissemination of study findings. The role of the YAP is to provide input and feedback to scientists working with YWH, alert scientists about pressing youth community issues and topics in HIV treatment and prevention, and assist scientists in disseminating research findings. The YAP will provide feedback on: (1) the schedule used to deliver incentives, (2) the size of the incentive, and (3) the type of incentive. YAP members also will provide input on the information that should be provided through the app and other study materials (e.g., surveys, instructions, videos). YAP members will review study materials and will provide input on recruitment, engagement, and retention strategies, next steps, and contextualization and dissemination of the findings.

### Recruitment and informed consent procedures

We will recruit YWH from AHF clinics in California and Florida. AHF will generate a list of YWH patients from their database who are age 18–29, speak English, are at least 3 months post-HIV diagnosis, and who are unsuppressed as defined by AHF as their most recent HIV viral load being over 20 copies per milliliter. From this list, patients will be contacted by AHF for interest in the study. All interested participants will be screened to confirm eligibility. If eligible, willing, and able to provide informed consent, participants will be enrolled in the study. Enrollment will occur online using DocuSign to consent and Qualtrics (Qualtrics, Provo, UT, USA; version March 2017) to complete the baseline and 3-month assessments. Participants will then be given instructions to download the app to their smartphone and follow the tutorial within the app to demonstrate how to take their daily dose with the app. During the consent conversation and follow-up conversations, participants will be encouraged to contact the research team if they have any additional questions.

### Consent procedures

Consent will occur online through DocuSign by study staff. Interested individuals will be given adequate time to read the consent form and the study staff will be available to answer any questions via phone or video-conferencing. The consent form will include information about potential risks and benefits, that participation is completely voluntary and will not affect the care that participants receive, and that they may withdraw from the study at any time. The individual must read and sign the informed consent before participating. As part of the consent form, participants will be notified that study staff will collect contact information to facilitate follow up but that this information will not be shared with others outside of the study. AHF staff will receive verbal or written approval from AHF clients who are interested in the study prior to sharing their information with UCSF study staff

### Intervention

AiCure (an artificial intelligence and advanced data analytics company) has developed an innovative NIH-funded app to evaluate medication-taking using aDOT. This technology can replace the need for other efforts to monitor adherence, which is often self-reported and retrospective. The AiCure app offers the advantages of detecting if the correct person is taking a medication at the correct time, providing real-time adherence data in the absence of provider/researcher visual confirmation, and recording the total amount of incentives earned. The AiCure app also addresses barriers common with other technologies, including worries or discomfort with recording videos of oneself, challenges with uploading videos to a secure website, and difficulties with scheduling a real-time video visit with a provider [14]. Further, this technology provides an evidence-based and innovative approach to remote, real-time adherence monitoring that is more accurate than self-reported adherence and reduces provider/researcher time and resources that are required by other similar apps.

**Fig 1:**
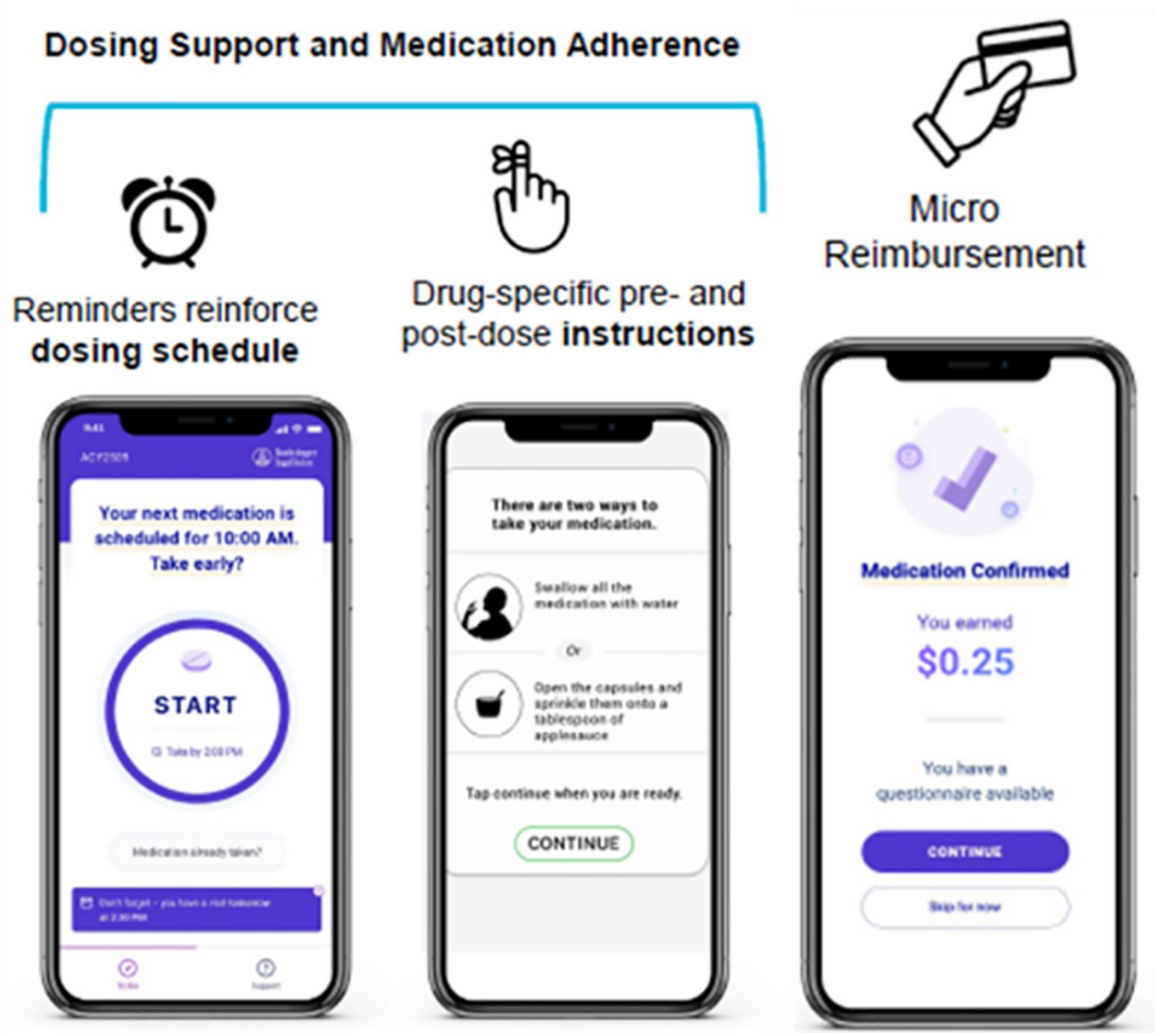
App features of AiCure app for directly observed therapy.

AiCure, which is Health Insurance Portability and Accountability Act (HIPAA)-compliant, is downloaded onto the participant’s smartphone as an app. Using facial recognition and computer vision, software algorithms track patient use, and confirm medication ingestion. This technology has been optimized over time through machine learning and annotation of the visual and audio data. AiCure’s facial recognition feature has been validated for accuracy with members of many racial and ethnic populations. The app confirms five steps of dosing: the participant’s face, the pill, pill in mouth/swallowing pill, participant showing an empty mouth and participant showing an empty under tongue area. In addition, participants receive daily dosing reminders that prompt them to open the app and complete aDOT or confirm that they have already taken their medication (Fig 1). Dosing data are encrypted and transmitted to a centralized, cloud-based system and displayed on a study dashboard to allow for real-time review and intervention by study staff. Each dosing event is recorded by the system and is given a date and time stamp. Participants can also self-report doses through a button within the app if they forget or are unable to use the system to confirm their dosing. Missed doses can be reclassified by the site through the dashboards as ‘self-reported’, if needed. Adherence data are grouped in the app as one of the following six categories: (1) visual confirmation of ingestion, (2) self-reported dose via the self-report button (no visual confirmation), (3) self-reported dose over the phone to the study staff, or (4) missed dose (no visual confirmation and no self-report). Suspicious activity or incorrect usage trigger alerts that are flagged for further review by an AiCure video support staff member such as intentional nonadherence (e.g., spitting out the medication or hiding it in the mouth without swallowing).

In the YEHE study, small financial incentives will also be tracked through the platform after each visually confirmed dose based on a small pre-set amount (e.g., $0.75 per dose). The amount of $0.75 was chosen as a starting point with which to pilot test the app and with feedback from the YAP. After correctly taking a dose, the participant will receive a message in the app saying that they have earned $0.75, and throughout the course of the study, the participants can use the “view earnings” button in the app to see their cumulative total awards earned. At the end of the week, the participant will receive the total weekly amount via Venmo or online gift card each month. In addition, if they have taken all 7 doses in one week, they will receive an additional incentive of $0.25 per day for a total of $1.00 per day. This incentive structure was chosen to address daily ART adherence and weekly ART persistence. This incentive structure was chosen based on feedback from the YAP. In our prior study using Venmo, 82% of participants chose to receive their incentives via Venmo and 18% choose to receive an electronic gift card [31]. The study will have a Venmo account and checks from the study will be put into the account. The account will use a study-specific mobile phone number and email, and will be accessible only by the study staff. Participants will be asked to provide their Venmo username, which will be verified. The default will be set to “private” so that participant information cannot be viewed by others upon payment.

### Participant retention and incentives

Participant retention will be enhanced through collection of detailed contact information, text messaging check-ins, and incentives. We will work with the YAP on maintaining and enhancing participant retention throughout the study. We will offer remuneration to all participants, which will be independent payments of $20 for completing the baseline survey, $50 for the 3-month survey, and $40 for completion of the in-depth interview. Some strategies that we have used to minimize loss-to-follow-up from research participation are to (1) involve YWH through the YAP; (2) collect extensive contact information at baseline to facilitate tracking, including names and information of 1–3 contacts who may know the participant’s whereabouts; (3) attain at least 3 methods of contact (e.g., phone number, email, and social media information); (4) provide gradually increasing yet non-coercive monetary incentives that encourages ongoing participation in study; (5) allow remote research participation to minimize barriers for participating; and (6) engage in monthly check-ins with participants to ask about app use and support logistical challenges with the app.

### Quantitative data collection and measures

YEHE will be a pilot study with 30 YWH who will use the platform for 3 months. Participants will complete online HIPAA-compliant Qualtrics surveys at baseline and 3 months. We will measure feasibility and acceptability through app paradata (i.e., app use information) and self-report in surveys (at baseline and 3 months; Tables 1 and 2; S1 Checklist). The primary outcomes are the feasibility and acceptability of the aDOT-CEI intervention. To determine if there is evidence to support a future trial, we will describe the percentage who meet each threshold for feasibility and acceptability at the end of the study period. We selected these thresholds (Table 1) based on our prior successful pilot studies among YWH [19, 32]. Demographic characteristics will also be collected and will include age, sex assigned at birth and gender identity, race, ethnicity, sexual orientation, education, income, work, school, living situation, city of residence, and ever homeless or incarcerated.

**Table 1:** Schedule of enrolment, interventions, and assessments*.

	STUDY PERIOD					
	Enrolment	Allocation	Post-allocation			Close-out
TIMEPOINT**	*-t*_*1*_	0	*t*_*1*_	*t*_*2*_	*t*_*3*_	*t*_*x*_
**ENROLMENT**:						
**Eligibility screen**	X					
**Informed consent**	X					
**Download aDOT-CEI App**	X					
***aDOT-CEI Intervention***		X	X	X	X	
**ASSESSMENTS**:						
***Demographics*, *use of technology***		X				
***Acceptability*, *feasibility*, *adherence***						X

*Recommended content can be displayed using various schematic formats. See SPIRIT 2013 Explanation and Elaboration for examples from protocols.

**List specific timepoints in this row.

**Table 2:** Outcome and descriptive variables.

Variables	Source	Threshold
**Process outcomes (Primary feasibility outcomes)**		
Retention by 3 months	Retention data; measured at 3-month survey	≥ 80% retention (i.e., 24 YWH retained)
Mean logins/week	App use data for time on task or time using app	≥ of 5 logins/week (i.e., ≥70% use/week)
Mean number of minutes in app	App use data for time on task or duration of time in app (min/day)	≥ of 1 minute in app/day
Intentional nonadherence	Artificial intelligence flagging the video to be confirmed by human review	Mean number of times participant may have “falsified” medication-taking (<20%)
**Client-level outcomes from exit survey (Primary acceptability outcomes)**		
Acceptability at 3 months	System Usability Scale (SUS; range 0–100) [33]	≥ 80% will have a score >68, considered above average and acceptable
Acceptability at 3 months	Client Satisfaction Questionnaire (CSQ-8) [34]	8 items; higher values indicate higher satisfaction / 80% have a score of ≥17
Recommend study to a friend	How likely are you to recommend the study to a friend? [32, 35]	7-point Likert Scale / ≥80% likely or very likely to recommend
Acceptability of intervention components	How satisfied were you with adherence monitoring, CEI, reminders, security and privacy, support? [32, 35]	7-point Likert Scale / ≥80% satisfied or very satisfied
**Clinical-level outcomes (Exploratory)**		
ART adherence	Adherence data from AiCure platform	Adherent: ≥80% of doses taken
		Non-adherent: <80% of doses taken
Self-reported ART adherence	Survey data. How much did participation in this study help you improve adherence to your medications? [32]	Adherent: no missed doses
	3-item self-reported measure of ART adherence [36]	Non-adherent: any missed doses
**Mechanisms of action from app paradata and exit survey**		
Monitoring of behavior	Number of minutes in app; How easy/difficult was it to use your personal phone; use adherence monitoring; receive incentives? [32, 35] Did you ever have trouble accessing app, using adherence monitoring, receiving reminders, receiving incentives, or finding a private place? [32, 35]	7-point Likert Scale / 80% say it’s easy or very easy to use.
Target behavior	Number of confirmed doses	-

Our sample size was chosen to provide variability in quantitative data while remaining feasible in scope for a pilot study evaluation of the intervention. Given that this is a pilot study, statistical significance testing will be de-emphasized; inferential analyses will be used to explore preliminary evidence of clinical impact and test measures and data collection protocols as a quality assurance step to ensure all measurement and analysis procedures can be successfully implemented in a subsequent formal efficacy trial. YEHE aims to assess the feasibility and acceptability of a novel aDOT-CEI intervention and is not designed or powered to determine the overall intervention effect nor the effects of individual components. Given the one-year timeline of this project, the recruitment of 30 YWH and provision of the AiCure app for three months is feasible.

### Qualitative measures

After the trial, we will conduct one-hour in-depth interviews (IDIs) with 15 YWH and 5 staff/providers purposively selected from participating AHF clinics based on their work with youth with HIV. Based on accepted norms for qualitative research, we anticipate that our sample size is sufficient to achieve theoretical saturation, but additional purposive sampling may be conducted to fill gaps, such as by high or low levels of adherence. Interviews will explore intervention experiences, potential influences on current and long-term ART adherence, unaddressed adherence barriers and the potential benefit of features (e.g., reminders), individual-level and clinic-level barriers and facilitators to intervention implementation, assess ease of use of aDOT-CEI, likes and dislikes, and suggested modifications for a future efficacy trial. We will also ask separately about experiences with the incentives versus the aDOT. Interviews will be conducted remotely using a HIPAA-compliant video-conferencing account with audio-recording. The interviews will be semi-structured to explore key questions, with allowance for iteration and probing on emergent themes raised by participants. We will explore the benefit of individual (aDOT, CEI) and combined components (aDOT+CEI). For qualitative in-depth interviews, study staff will obtain verbal informed consent either by phone or video-conferencing, and consent documents will be emailed to participants. Any and all participant questions and concerns will be addressed prior to beginning the interview.

### Data management and storage

An electronic database system (stored on a HIPAA-compliant server) will be used to monitor participants’ progress through the study, and participants’ adherence will be monitored via the AiCure app. All files will be password-restricted and user ID protected so that only research staff will have access to the files. Data will be collected solely for this study. Loss of confidentiality is the main potential risk associated with participation in this study. We will take precautions to minimize loss of confidentiality. Identifying information will be stored separately from health and behavioral information. Health and behavioral data will be identified only with a participant ID. Individuals will not be identified in any reports or publications of the research. All participant-identifying data maintained for the study will be accessible only to the project staff via the AiCure app or Qualtrics, and via a secure server that is only accessible to selected study staff.

### Security and confidentiality

The patient portal seen by the research coordinator is secure and de-identified. The app icon is study and treatment agnostic and does not contain any symbol or name that would identify someone as living with HIV or as a member of the study, so that anyone who may inadvertently see the icon on a participant’s smartphone cannot trace it to this study. Any participant who does not feel comfortable answering any survey questions will be reminded that study participation is voluntary and they may skip any questions. Participants will be reminded that they may withdraw from the study at any point.

In qualitative interviews, to protect against loss of privacy and confidentiality, prior to initiating the audio-recording, participants will be asked to not use any names and all names stated inadvertently will be replaced with aliases in transcripts. All interviews will be conducted via a HIPAA-compliant video-conferencing platform (e.g., Zoom) and audio-recordings will be stored on a HIPAA-compliant server with access available only to select study staff. All audio-recorded files will be deleted on completion of the study. All data will be collected for the purpose of this research only and will be password protected.

### Planned analyses

#### Quantitative analysis

The primary outcomes are feasibility and acceptability of the aDOT-CEI intervention. To assess these outcomes, we will describe feasibility and acceptability measures at each time point. One-way frequency tables will be generated for all feasibility and acceptability measures and measures of central tendency and variability will be computed for continuous measures. To examine feasibility of intervention procedures, we will examine demographic characteristics of those who participated in the study, and who were retained as compared with those who were not, using two-way tables. To determine if there is evidence to support a future trial, we will describe the percentage who meet each threshold for feasibility and acceptability (Table 1). We will examine change in self-reported adherence through the survey and adherence monitored through the app over time but this will be exploratory given the small sample size.

#### Qualitative analysis

The research coordinator will transcribe and de-identify all interviews by removing names and identifying details and assist with analysis using qualitative analysis software. Qualitative software allows for excerpts of transcribed interviews to be coded, identified, and associated with different themes from the interviews. This will allow the analysis to organize content into higher-order themes while also allowing easy access to personal narratives of the respondents. We will develop a codebook and organize themes on the basis of the interview excerpts and discuss and utilize analytic memos. During analysis, we will draw on our knowledge of the intervention and the Key Principles in Contingency Management Implementation [28] to determine how participant feedback may shape future iterations of the aDOT-CEI.

### Ethics and dissemination

We received approval from the UCSF Institutional Review Board (IRB) to conduct this study and require written consent from all participants prior to enrollment. We plan to disseminate the research results widely to researchers, clinicians, and community members. At the conclusion of the project, we will share study findings through the YAP, UCSF Division of Prevention Science, AHF, and RTI. Dissemination through AHF will be used to solicit input on the findings from AHF staff and providers to guide modifications and use in a future effectiveness trial. We will also share the findings with other interested parties to determine next steps. Additionally, we will work with the UCSF Community Engagement Core and the UCSF Prevention Research Center to ensure that our findings are distributed widely, posted on websites, and on social media accounts through Twitter, LinkedIn, Facebook, and YouTube.

### Status and timeline of the study

Recruitment began in February 2023, and we have enrolled 29 participants as of June 2023. We expect to complete 3 months of follow-up data collection by August 2023.

## Discussion

Our study is significant because it has the potential to be effective while improving ART adherence and virologic suppression among a key population experiencing health disparities, YWH. This research builds on our prior work using technology-based interventions to improve HIV clinical outcomes among YWH [19, 32, 37–41]. Our intervention is innovative because we will use a novel aDOT-CEI intervention among YWH who are not virologically suppressed. This project will document the feasibility and acceptability of aDOT-CEI and will explore preliminary data to test the efficacy of aDOT-CEI in measuring adherence and addressing disproportionately low viral suppression among YWH.

YWH have poor HIV outcomes across the care continuum including increased ART nonadherence and virologic failure [1, 2]. Tracking and monitoring objective measures of ART adherence in real time is critical to strategies to support adherence and improve clinical outcomes for this population. To our knowledge, no research to date has used aDOT to improve HIV outcomes among YWH and only one study has examined an HIV outcome in relation to digital directly observed therapy [14, 18]. aDOT is evidence-based and is an innovative approach to remote, real-time adherence monitoring that is both more accurate than self-reported adherence and reduces provider/researcher burden above what is provided by other similar apps [16, 17, 29]. Rather than collecting written adherence logs that providers must interpret, or requiring live or video directly observed therapy, AiCure is able to uniquely interact with patients who are taking medications. It discretely and easily guides and encourages them to properly take medications and can notify healthcare or research personnel when patients do not take their medications. aDOT also may reduce provider burden by allowing them to focus more time and effort on subpopulations that need them most. Further, our prior research has shown that self-reported adherence is correlated with other objective remotely collected adherence measures indicating that remote, technology-based solutions may be important and useful for measuring adherence [42]. Artificial intelligence technology is expected to grow and become ubiquitous across healthcare fields and will become less expensive over time as it becomes more widely adopted [43]. This technology will advance adherence monitoring and can easily be incorporated into routine care to identify individuals in need of support. This study will provide important data on feasibility and acceptability of monitoring medication-taking using a real-time objective measure of adherence based on a smartphone application that uses facial recognition with artificial intelligence.

In addition to providing data on the use of aDOT to measure adherence in a novel population, this study combines aDOT with technology-delivered CEIs to improve HIV outcomes. To our knowledge, no research to date has combined aDOT with other evidence-based interventions to incentivize adherence. CEI has been used successfully among young people to increase PrEP use and in combination with in-person DOT, but not with digital DOT [28, 44–46]. aDOT records adherence through facial recognition and has been used to provide financial incentives for clinical trial participation but not for behavior change strategies. The aDOT platform, with its real-time adherence monitoring data, provides an ideal platform by which to immediately incentivize positive behavior conditional on ART adherence levels. Incentives will be in closer proximity to the behavior via the real-time information on adherence, which will have a stronger effect on behavior change [28]. CEI addresses common barriers to adherence among YWH by offsetting the cost of seeking health services and increasing motivation in young people, and may therefore further bolster the effects of increased monitoring provided by aDOT.

There are several limitations to our study. Given that over 96% of individuals ages 18–29 in the US have a smartphone [11], we are confident that the vast majority of our target population will have a smartphone. However, we acknowledge that there will be a small subset of individuals who may not have smartphone access (~4%) and for whom the results of this study may not be generalizable. Additionally, YWH are able to self-report adherence in the app if they chose to not use the facial recognition components. This may cause incorrect self-reported data due to social desirability bias. We are unable to confirm self-reported adherence. Lastly, the sample size of the study is small, which may result in wide interval estimates limit generalizability. The estimates obtained in this pilot study will not be used as inputs for a future formal efficacy trial; instead, those inputs will be based on pre-determined clinically meaningful differences informed by the published literature that will be available at that time.

In lieu of a gold standard for estimating ART adherence, aDOT is an objective, easy-to-use, low-burden, and potentially time- and cost-effective option for adherence monitoring in the United States and other countries with widespread use of technology [12, 47]. Digital DOT is not subject to many biases of self-reported measures, is non-invasive (no need for cutting hair, pricking fingers, etc.), provides real-time data and is HIV sero-neutral or can be used by studies that are recruiting for both HIV treatment and prevention can use a unified measure (which may not be possible for biomarkers). Further, digital DOT is more cost-effective than in-person DOT [12, 14]. Incentives have been effective at increasing adherence, have been used at Ryan White facilities [48], and may be more cost-effective than poor clinical outcomes. If we can demonstrate that the intervention is acceptable and feasible, we will have obtained valuable data to inform the design a larger, fully powered R01 study to evaluate efficacy. If found to be effective, the aDOT-CEI intervention can be scaled up as an important cost-effective and objective approach to measuring adherence and achieving optimal reach and impact of ART among YWH who are disproportionately impacted by HIV and at elevated risk for poor clinical outcomes.

## Supporting information

S1 Checklist SPIRIT 2013 checklist: Recommended items to address in a clinical trial protocol and related documents. (DOC)

S1 File (DOCX)
