# Supplementary material for: Youth Ending the HIV Epidemic (YEHE): Protocol for a pilot of an automated directly observed therapy intervention with conditional economic incentives among young adults with HIV
Source: PLoS One. 2023 Dec 22;18(12):e0289919. doi: 10.1371/journal.pone.0289919 (PMC10745168; doi:10.1371/journal.pone.0289919)
Supplement: S1 File — (DOCX) [file pone.0289919.s002.docx]

YEHE Study Protocol

IRB Study Number: 22-36721

Version #1, Date: 7/2023

Contents

[Protocol Title 1](#_Toc140846484)

[Background 1](#_Toc140846485)

[Objectives 2](#_Toc140846486)

[Preliminary Studies 3](#_Toc140846487)

[Sample Size and Eligibility 3](#_Toc140846488)

[Recruitment 4](#_Toc140846489)

[Procedures 4](#_Toc140846490)

[Consent Process 6](#_Toc140846491)

[Data Collection and Analysis 6](#_Toc140846492)

[Risks to Subjects 8](#_Toc140846493)

[Risk to Benefit Ratio 9](#_Toc140846494)

[Data Management 10](#_Toc140846495)

[References 10](#_Toc140846496)

# Protocol Title

Youth Ending the HIV Epidemic - Automated Directly Observed Therapy Pilot: Improving HIV

Care Among Youth

# Background

**Health disparities among young adults living with HIV (YLWH)**. Despite improvements in life expectancy among people living with HIV (PLWH) and reductions in HIV transmission with increased availability of antiretroviral therapy (ART), a sustained effect of ART depends on high levels of adherence. In the US in 2018, for every 100 youth diagnosed with HIV, 79 received some HIV care, 58 were retained in care, and 60 were virally suppressed.^1^ These figures are dramatically lower than those for older adults.^2-5^ In addition to increased onward HIV transmission, poor adherence can increase drug resistance, leading to less effective treatment options and worse clinical outcomes.^6^ Consequently, YLWH represent a priority population to address with efforts to increase re-engagement in care and improved ART adherence. The overarching goal of this Ending the HIV Epidemic (EHE) Supplement application is to pilot test an innovative intervention, Youth Ending the HIV Epidemic (YEHE), to improve ART adherence and HIV virologic suppression in 18–29-year-old YLWH.

**Use of an innovative intervention to improve HIV health outcomes among YLWH**. There is growing interest in the use of novel technologies to provide objective measures to efficiently monitor and support medication adherence with reduced cost and burden to patients and providers. These tools are well suited for young people who are proportionately high users of mobile technologies.^7-9^ Digital directly observed therapy (DOT) has been shown to be acceptable, feasible, and effective at increasing medication adherence in different populations and for a range of health outcomes, including adherence to HIV pre-exposure prophylaxis (PrEP).^10-14^ Automated DOT (aDOT) is an innovative technology that uses artificial intelligence (AI) with computer vision and deep learning algorithms to track and support adherence through a smartphone.^10,15-17^ However, to our knowledge, aDOT has not been used to help enhance ART adherence among YLWH who may need adherence support. Additionally, aDOT provides a seamless and convenient platform for providing Conditional Economic Incentives (CEIs) because it monitors real-time adherence to automatically determine who can receive incentives. CEIs use operant conditioning to reinforce behavior through financial incentives or prizes^18^ that have been shown to be sustainable in some settings.^19^ CEI has been used successfully to increase ART adherence and promote behavior change, and studies using in-person DOT for tuberculosis have found that the benefits of DOT were even further increased with the addition of financial incentives.^20-25^ Consequently, a combined aDOT-CEI platform where medication-taking is monitored and incentivized in real time is a promising approach to increase ART adherence in YLWH. We aim to pilot test an aDOT-CEI intervention, YEHE, to improve ART adherence among YLWH (18–29 years-old) in California and Florida who have an unsuppressed HIV viral load.

Our study is significant because it has the potential to be cost-effective while improving ART

adherence and virologic suppression among a key population experiencing health disparities. This research builds on our prior work using technology-based interventions to improve HIV clinical outcomes among YLWH.^26-29^ Our intervention is innovative because we will use a novel aDOT-CEI intervention among YLWH who are not virologically suppressed. This EHE application will document the feasibility and acceptability of aDOT-CEI and will provide preliminary data to inform an R01 to test the efficacy of aDOT-CEI in addressing disproportionately low viral suppression among YLWH.

# Objectives

Young adults have a disproportionately high rate of HIV infection, high rates of attrition at all stages of the HIV care continuum, an increased risk of antiretroviral therapy (ART) nonadherence and virologic failure, and a high probability of disease progression and transmission. Tracking and monitoring objective measures of ART adherence in real time is critical to strategies to support adherence and improve clinical outcomes. However, adherence monitoring often relies on self-reported and retrospective data or requires extra effort from providers to understand adherence patterns,^30^ making it difficult for providers to accurately determine how to support their patients in real time. In the proposed study, we will monitor medication-taking using a real-time objective measure of adherence that does not rely on healthcare providers for assessment.

Prior research shows high feasibility and acceptability of technology-based directly observed therapy (DOT) to improve medication adherence with reduced provider and patient burden versus in-person DOT.^10-14^ Additionally, financial barriers, side effects, and struggles with waning motivation to continue ART are commonly cited barriers to adherence among young adults living with HIV (YLWH).^31,32^ Conditional Economic Incentives (CEIs) have been used successfully to promote a range of positive behaviors and have specifically been coupled with in-person DOT to improve ART adherence.^18,20,21^ In the current proposal, we build on the existing evidence and interventions and address underlying gaps in research by aiming to pilot test the Youth Ending the HIV Epidemic (YEHE) intervention to improve ART adherence in 18–29-year-old YLWH in California and Florida.

In Aim 1, we will enroll YLWH aged 18 to 29 to participate in a 3-month pilot study to assess the feasibility and acceptability of a novel automated directly observed therapy-conditional economic incentive (aDOT-CEI) intervention. In Aim 2, some of the pilot participants and staff/providers from participating AIDS Healthcare Foundation (AHF) clinics will participate in individual in-depth qualitative interviews to explore intervention and implementation facilitators and barriers. These Aims will provide quantitative and qualitative evidence on feasibly and acceptability to determine whether a future trial is merited based on set criteria.

# Preliminary Studies

Prior research shows high feasibility and acceptability of technology-based DOT to improve

medication adherence with reduced provider and patient burden versus in-person DOT.^10-14^

Additionally, financial barriers, side effects, and struggles with waning motivation to continue ART are commonly cited barriers to adherence among YLWH.^31,32^ CEIs have been used successfully to promote a range of positive behaviors and have specifically been coupled with in-person DOT to improve ART adherence.^18,20,21^ In the current proposal, we build on the existing evidence and interventions and address underlying gaps in research by aiming to pilot test a novel mHealth app that combines aDOT with CEI to improve ART adherence in 18–29-year-old YLWH.

# Sample Size and Eligibility

***Total Enrollment*** – 30 participants and 5 providers/staff from the AHF clinics

- Aim 1: 30 YLWH
- Aim 2: 15 YLWH from Aim 1 + 5 providers/staff from the AHF clinics

***Inclusion Criteria –*** Participants will be included if they meet the following

criteria:

- 18 to 29 years of age
- Have access to a smartphone
- Can speak and read English or Spanish
- Client is at an AHF clinic in California or Florida
- Client has an unsuppressed HIV viral load at least 3 months post HIV diagnosis
- Client is on once daily oral antiretroviral therapy
- Consent to participate in the proposed study

***Exclusion Criteria -*** Participants will be excluded if they meet the following

criteria:

- YLWH who are newly HIV diagnosed in the past 3 months
- Smartphone is a Samsung galaxy s21 or iPhone 5SE (app is not supported by these phones)

# Recruitment

We will recruit YLWH from AHF clinics in CA (San Francisco, Oakland, Los Angeles, and San Diego) and FL. AHF will generate a list of clients from their database of YLWH aged 18–29 who have an unsuppressed viral load. From this list, clients will be randomly selected and contacted by AHF for interest in the study (refer to attached recruitment script). To recruit YLWH who are out of care, clients will be contacted by AHF providers or staff through the Back in Care program. Clients out of care are called regularly and will be offered the intervention to assess interest and eligibility. Interested clients will be encouraged to contact the study team for further information, screening, and enrollment; or they may be asked for permission to share their contact information with the study team to reach out to them (refer to attached screening script).

Clients may also reach out to the study team directly if they see a flyer in the clinic.

For Aim 2, a sample of the YLWH participants and staff/providers from participating AHF clinics will be purposively selected and invited to participate in an interview. Research staff will reach out by phone/text/email to assess for interest and schedule a time for the interview.

# Procedures

In Aim 1, we will enroll YLWH aged 18 to 29 to participate in a 3-month pilot study to assess the feasibility and acceptability of a novel automated directly observed therapy-conditional economic incentive (aDOT-CEI) intervention. In Aim 2, some of the pilot participants and staff/providers from participating AIDS Healthcare Foundation (AHF) clinics will participate in individual in-depth qualitative interviews to explore intervention and implementation facilitators and barriers. These Aims will provide quantitative and qualitative evidence on feasibly and acceptability to determine whether a future trial is merited based on set criteria.

In the first year of the study, we will hire and train study staff. We will invite YLWH from AHF sites in CA to form the study Youth Advisory Panel (YAP) and seek their input throughout the study on the AiCure app used for the intervention, financial incentive structure, recruitment and retention strategies, and dissemination of the study findings. The YAP will provide feedback on (1) the schedule used to deliver incentives, (2) the size of the incentive, and (3) the type of incentive. YAP members also will provide input on the information that should be provided through the app and other study materials (e.g., communication about how to use the app to receive incentives, and reminder messages). YAP members will review study materials (surveys, instructions, videos) before study launch and will provide input on recruitment, engagement, and retention strategies, next steps, and contextualization and dissemination of the findings. We will work with AiCure to implement any required changes to the app that have emerged from formative research. The app will then be piloted with YLWH who will use the platform for a period of 3 months (Aim 1).

For the pilot, we will recruit YLWH from AHF clinics in CA and FL. During enrollment, participants will be informed about the study. Following consent, participants will be given instructions to download the app to their mobile phone, and study staff will demonstrate how to use the app via a remote conferencing platform. They will complete a tutorial and demonstrate taking ART using the app. The app will record video of the participant taking their HIV medication in order to monitor the participant's medication adherence. Participants will complete online surveys at baseline and 3 months. We will have monthly check-ins with participants which we will assess app use and help increase study engagement. We will measure feasibility and acceptability through app paradata (i.e., app use information) and self-report in surveys (baseline, 3 months). At consent, we will also have the participants sign a HIPAA form so that we may request adherence related medical record data from their AHF clinic. We will use this data to compare against adherence monitored by the AiCure app.

Following completion of the pilot, we will conduct in-depth interviews (IDIs) with YLWH and staff/providers purposively selected from participating AHF clinics. Interviews will explore intervention experiences, potential influences on current and long-term ART adherence, unaddressed adherence barriers and the potential benefit of features (e.g., reminders), individual-level and clinic-level barriers and facilitator to intervention implementation, assess ease of use of aDOT-CEI, likes and dislikes, and suggested modifications for a future efficacy trial. Interviews will be conducted remotely using a HIPAA-compliant, licensed Zoom account and audio-recorded. The interviews will be semi-structured to explore key questions, with allowance for iteration and probing on emergent themes raised by participants. We will explore the benefit of individual (aDOT, CEI) and combined components (aDOT+CEI).

During the study, we will continue to meet with the YAP to discuss challenges and potential solutions. After completion of the study, we will discuss the study findings with the YAP and other groups, such as the Center for AIDS Prevention Studies (CAPS), the CAPS Community Advisory Board (CAPS CAB), Departments of Public Health in major cities of California, California State Office of AIDS, Medi-Cal managed care plans, private insurers (such as Kaiser Permanente), Ryan White planning councils, and other groups with a strong working knowledge of HIV service delivery and funding in California. The purpose of these meetings will be to determine next steps and developing sustainable models for implementation of this service should it be found to be effective. For example, if the study is efficacious, we will explore mechanisms for its implementation.

# Consent Process

Aim 1: Interested clients will be encouraged to contact the study team, or will be asked for

permission to share their contact information with the study team. The study team will screen

them to determine eligibility, and if eligible, will offer to enroll them in the study. Consent and

enrollment will occur online through Qualtrics by a study team member. Interested individuals will be given adequate time to read the consent form and the study team member will be available to answer any questions via phone. The consent form will include information about potential risks and benefits, that participation is completely voluntary and will not affect the care that participants receive, and that they may withdraw from the study at any time. The individual must read and sign the informed consent before participating. As part of the consent form, participants will be notified that we will collect extensive contact information to facilitate tracking, including (1) names and information of 1 to 3 contacts who may know the participant’s whereabouts; (2) collection of additional information, including addresses of sites that the participant frequents, such as free food distribution centers, shelters, bars, probation/parole offices, etc.; and (3) attaining at least 3 methods of contact (e.g., mobile telephone number, email address, mailing address, or social media username).

Aim 2: Study staff will obtain verbal informed consent either by phone or Zoom, and consent

documents will be emailed to participants. Any and all participant questions and concerns will be addressed prior to beginning the interview.

# Data Collection and Analysis

In Aim 1, we will conduct a pilot study to assess feasibility and acceptability of the use of aDOT-CEI among 30 YLWH and collect survey data at baseline and after 3 months of app use (**Table 1**). We will measure feasibility and acceptability through app paradata (i.e., app use information) and self-report in surveys (baseline, 3 months).

| **Table 1.** Outcome and Descriptive Variables. | | |
| --- | --- | --- |
| **Variables** | **Source** | **Threshold** |
| **Process outcomes (Primary feasibility outcomes)** | | |
| Retention by 3 months | Retention data; measured at 3-month survey | ≥ 80% retention (i.e., 24 YLWH retained) |
| Mean logins/week | App use data for time on task or time using app | ≥ of 5 logins/week (i.e., ≥70% use/week) |
| Mean number of minutes in app | App use data for time on task or duration of time in app (min/day) | ≥ of 1 minute in app/day |
| Intentional nonadherence | AI flagging the video to be confirmed by human review | Mean number of times participant may have “falsified” medication-taking (<20%) |
| **Client-level outcomes from exit survey (Primary acceptability outcomes)** | | |
| Acceptability at 3 months | System Usability Scale (SUS; range 0–100)^101^ | ≥ 80% will have a score >68, considered above average and acceptable |
| Acceptability at 3 months | Client Satisfaction Questionnaire (CSQ-8)^102^ | 8 items; higher values indicate higher satisfaction / 80% have a score of ≥17^103^ |
| Recommend study to a friend | How likely are you to recommend the study to a friend?^29,31^ | 7-point Likert Scale / ≥80% likely or very likely to recommend |
| Acceptability of intervention components | How satisfied were you with adherence monitoring, CEI, reminders, security and privacy, support?^29,31^ | 7-point Likert Scale / ≥80% satisfied or very satisfied |
| **Clinical-level outcomes (Exploratory)** | | |
| ART adherence | Adherence data from AiCure platform | Adherent: ≥80% of doses taken  Non-adherent: <80% of doses taken |
| Self-reported ART adherence | Survey data. How much did participation in this study help you improve adherence to your medications?^29^  3-item self-reported measure of ART adherence^104^ | Adherent: no missed doses  Non-adherent: any missed doses |
| **Mechanisms of action from app paradata and exit survey** | | |
| Monitoring of behavior | Number of minutes in app; How easy/difficult was it to use your personal phone; use adherence monitoring; receive incentives?^29,31^ Did you ever have trouble accessing app, using adherence monitoring, receiving reminders, receiving incentives, or finding a private place?^29,31^ | 7-point Likert Scale / 80% say it’s easy or very easy to use. |
| Target behavior | Number of confirmed doses | - |

In Aim 2, we will explore intervention and implementation facilitators/barriers by conducting in-depth qualitative interviews with 15 YLWH and 5 staff/providers from participating AHF clinics. We will examine their intervention experiences, potential influences on current and long-term ART adherence, unaddressed adherence barriers and potential benefit of features (e.g., reminders), individual-level and clinic-level barriers and facilitators to intervention implementation, assess ease of use of aDOT-CEI, and suggested modifications for a future efficacy trial.

The proposed project aims to assess the feasibility and acceptability of a novel aDOT-CEI intervention and is not designed or powered to determine the overall intervention effect nor the effects of individual components.^33-37^ It will provide quantitative and qualitative evidence on feasibly and acceptability to determine whether a future trial is merited based on set criteria.

**Quantitative analysis**. The primary outcomes are feasibility and acceptability of the aDOT-CEI

intervention. To assess these outcomes, we will describe feasibility and acceptability measures at each time point within the intervention arm. One-way frequency tables will be generated for all feasibility and acceptability measures and measures of central tendency and variability will be computed for continuous measures. To examine feasibility of intervention procedures, we will examine demographic characteristics of those who participated in the study as compared with those who did not, and who were retained as compared with those who were not, using two-way tables. Demographic characteristics will include age, sex/gender, race/ethnicity, sexual identity, education, income, work, school, living situation, city of residence, and ever homeless or incarcerated. To determine if there is evidence to support a future trial, we will describe the percentage who meet each threshold for feasibility and acceptability at the end of the study period. We selected these thresholds based on our prior pilot studies among YLWH.^27,29^ Mechanisms of action will be assessed in formative research and through qualitative and quantitative data to inform any needed modifications to implementation for a future trial.

**Qualitative analysis**. The research coordinator will transcribe and de-identify all interviews by removing names and identifying details, and assist with analysis using Dedoose qualitative analysis software. Dedoose software allows for excerpts of transcribed interviews to be coded to be identified and associated with different themes from the interviews. This will allow the analysis to organize content into higher-order themes while also allowing easy access to personal narratives of the respondents. We will develop a codebook and organize themes on the basis of the interview excerpts and discuss and utilize analytic memos.^38^ During analysis, we will draw on our knowledge of the intervention and the Key Principles in Contingency Management Implementation^23^ to determine how participant feedback may shape future iterations of the aDOT-CEI.

# Risks to Subjects

**Protection against loss of privacy:**

Loss of confidentiality is the main potential risk associated with participation in this study. We will take appropriate precautions to minimize loss of confidentiality. Identifying information will be stored separately from health and behavioral information. Health and behavioral data will be identified only with a participant ID. Individuals will not be identified in any reports or publications of the research. All participant identifying data maintained for the study will be accessible only to the project staff via the aDOT mobile health app or Qualtrics, which are HIPAA-compliant, and via a HIPAA-compliant server that is only accessible to selected project staff.

Security and privacy are our top priority. Consequently, for the design and development of the mobile health app, we have partnered with AiCure to use an existing HIPAA-compliant mobile health app that maximizes security. Participants are automatically logged off each time they exit the app. The patient portal seen by the Research Coordinator is secure and de-identified. The app icon is study and treatment agnostic and does not contain any symbol or name that would identify someone as being HIV-positive or as a member of the study, so that anyone who may inadvertently see the icon on a participant’s mobile phone cannot trace it to this study.

Founded in 2010 and funded by the National Institutes of Health (NIH) and leading institutional investors, AiCure has more than 65 issued patents and works with global clients in over 40 countries. The AiCure aDOT mobile health app has been used in numerous populations to improve adherence to medication and validated with individuals of different races/ethnicities. AiCure will continue to update and improve the app within the known best practices for HIPAA-compliant apps and will maintain the highest level of security.

Any participant who does not feel comfortable answering any survey questions at baseline and 3 months will be reminded that study participation is voluntary and that they may withdraw at any time. Participants will be reminded that they may withdraw from the study at any point and not have their clinical information transferred from AHF and incorporated in the study database.

In Aim 2, to protect against loss of privacy and confidentiality, prior to initiating the audio recording of the qualitative interviews, participants will be asked to not use any names and all names stated inadvertently will be replaced with aliases in transcripts. All these interviews will be conducted and audio-recorded via a HIPAA-compliant web conference platform (e.g., Zoom) and audio-recordings will be stored on a HIPAA-compliant server with access available only to select study staff who are not employed at AHF (to protect employee participant anonymity). All audio recorded files will be deleted on completion of the study. All data will be collected for the purpose of this proposed research only and will be password protected.

Dr. Saberi will consult with Drs. Stoner and Sukhija-Cohen on a weekly basis for specific problems and questions as they may arise. Drs. Saberi, Stoner, Neilands, and Sukhija-Cohen have been involved in prior large-scale intervention and assessment trials that included vulnerable participants and confidential personal information. Dr. Saberi will consult with the UCSF Institutional Review Board (IRB) for guidance throughout the course of the study..

# Risk to Benefit Ratio

Participants may potentially benefit through an improved ability to engage in HIV care (including increased retention, enhanced ART adherence, and achievement of viral suppression), CEIs, and study-participation incentives. Improving engagement in care could lead to improved quality of life and reduced HIV transmission to others. If the study finds that the intervention is feasible and acceptable, the intervention could be expanded further to a large multisite randomized clinical trial to improve engagement in HIV care among YLWH, other age groups living with HIV, and other conditions where medication adherence is critical (e.g., hepatitis C treatment).

The proposed study will provide important information about the feasibility and acceptability of an aDOT-CEI intervention to increase viral suppression among YLWH. This information is important for research among YLWH who may have greater difficulty adhering to their medications and experience health disparities at a higher rate than older individuals living with HIV. The information gained could influence and improve care for other YLWH. Consequently, the minor potential risks to participants are reasonable in relation to the importance of the knowledge to be gained.

# Data Management

All data collected during the study will be used for research purposes only. The sources of data

will be mainly paradata from the AiCure app, self-report from participants on online surveys,

qualitative interviews, and clinical data provided by AHF. No blood specimens or other body fluid will be obtained for this study, and no hazardous conditions are imposed in the conduct of this study. The sources of research materials for this proposed study are data from the following:

- The AiCure platform and app usage data (paradata). We have received approval to use this app from the IT security assessment team.
- Online survey data from youth participating in our research (collected at baseline and 3 months), which include demographic data, healthcare accessibility, technology use, perceived engagement in HIV care, engagement with provider, and subsistence needs.
- AHF clinical data (collected as part of health care), including HIV viral load, appointment attendance and antiretroviral therapy adherence.
- Qualitative interviews where YLWH and staff/providers from participating AHF clinics discuss their experience with and their perspectives on their HIV care and on participating in this study. Qualitative interviews will include questions around intervention experiences, potential influences on current and long-term ART adherence, unaddressed adherence barriers and the potential benefit of features (e.g., reminders), individual-level and clinic level barriers and facilitator to intervention implementation, assess ease of use of aDOTCEI, likes and dislikes, and suggested modifications for a future efficacy trial.

The research team will have access to participant identities. Data will be collected solely for this

study. An electronic database system (stored on a Health Insurance Portability and Accountability Act of 1996 [HIPAA]-compliant server) will be used to track participants, and participants also will be tracked via the aDOT mobile health app, which is also HIPAA-compliant. All files will be password restricted and user ID protected so that only certain identified research staff will have access to the files. All data will be electronically collected and stored on HIPAA-compliant servers and will be limited to study staff only.

# References

1. Centers for Disease Control and Prevention. HIV and Youth: Viral Suppression. 2021; https://www.cdc.gov/hiv/group/age/youth/viral-suppression.html. Accessed March 9, 2022.

2. Chen M, Rhodes PH, Hall IH, et al. Prevalence of undiagnosed HIV infection among persons aged >/=13 years--National HIV Surveillance System, United States, 2005-2008. MMWR Morbidity and mortality weekly report. 2012;61 Suppl:57-64.

3. Torian LV, Wiewel EW, Liu KL, Sackoff JE, Frieden TR. Risk factors for delayed initiation of medical care after diagnosis of human immunodeficiency virus. Arch Intern Med. 2008;168(11):1181-1187.

4. Rutstein RM, Gebo KA, Flynn PM, et al. Immunologic function and virologic suppression among children with perinatally acquired HIV infection on highly active antiretroviral therapy. Med Care. 2005;43(9):15-22.

5. Zanoni BC, Mayer KH. The Adolescent and Young Adult HIV Cascade of Care in the United States: Exaggerated Health Disparities. Aids Patient Care St. 2014;28(3):128-135.

6. Kim SH, Gerver SM, Fidler S, Ward H. Adherence to antiretroviral therapy in adolescents living with HIV: systematic review and meta-analysis. Aids. 2014;28(13):1945-1956.

7. Lee M, Lee H, Kim Y, et al. Mobile App-Based Health Promotion Programs: A Systematic Review of the Literature. International Journal of Environmental Research and Public Health. 2018;15(12).

8. Gold J, Lim MSC, Hocking JS, Keogh LA, Spelman T, Hellard ME. Determining the Impact of Text Messaging for Sexual Health Promotion to Young People. Sex Transm Dis. 2011;38(4):247-252.

9. Mulawa MI, LeGrand S, Hightow-Weidman LB. eHealth to Enhance Treatment Adherence Among Youth Living with HIV. Current HIV/AIDS reports. 2018;15(4):336-349.

10. Liu AY, Laborde ND, Coleman K, et al. DOT Diary: Developing a Novel Mobile App Using Artificial Intelligence and an Electronic Sexual Diary to Measure and Support PrEP Adherence Among Young Men Who Have Sex with Men. Aids Behav. 2021;25(4):1001-1012.

11. Macaraig M, Lobato MN, Pilote KM, Wegener D. A National Survey on the Use of Electronic Directly Observed Therapy for Treatment of Tuberculosis. Journal of Public Health Management and Practice. 2018;24(6):567-570.

12. Garfein RS, Doshi RP. Synchronous and asynchronous video observed therapy (VOT) for tuberculosis treatment adherence monitoring and support. J Clin Tuber Oth Myc. 2019;17.

13. Ngwatu BK, Nsengiyumva NP, Oxlade O, et al. The impact of digital health technologies on tuberculosis treatment: a systematic review. Eur Respir J. 2018;51(1).

14. Parmar P, Mackie D, Varghese S, Cooper C. Use of Telemedicine Technologies in the Management of Infectious Diseases: A Review. Clin Infect Dis. 2015;60(7):1084-1094.

15. Shiovitz TM, Bain EE, McCann DJ, et al. Mitigating the Effects of Nonadherence in Clinical Trials. J Clin Pharmacol. 2016;56(9):1151-1164.

16. Ernsting C, Dombrowski SU, Oedekoven M, et al. Using Smartphones and Health Apps to Change and Manage Health Behaviors: A Population-Based Survey. Journal of medical Internet research. 2017;19(4).

17. Labovitz DL, Shafner L, Gil MR, Virmani D, Hanina A. Using Artificial Intelligence to Reduce the Risk of Nonadherence in Patients on Anticoagulation Therapy. Stroke. 2017;48(5):1416-+.

18. Galarraga O, Genberg BL, Martin RA, Laws MB, Wilson IB. Conditional Economic Incentives to Improve HIV Treatment Adherence: Literature Review and Theoretical Considerations. Aids Behav. 2013;17(7):2283-2292.

19. de Walque D. The use of financial incentives to prevent unhealthy behaviors: A review. Soc Sci Med. 2020;261.

20. Volmink J, Matchaba P, Garner P. Directly observed therapy and treatment adherence. Lancet. 2000;355(9212):1345-1350.

21. Davidson H, Schluger NW, Feldman PH, Valentine DP, Telzak EE, Laufer FN. The effects of increasing incentives on adherence to tuberculosis directly observed therapy. International Journal of Tuberculosis and Lung Disease. 2000;4(9):860-865.

22. Petry NM, Rash CJ, Byrne S, Ashraf S, White WB. Financial Reinforcers for Improving Medication Adherence: Findings from a Meta-analysis. American Journal of Medicine. 2012;125(9):888-896.

23. Stanger C, Budney AJ. Contingency Management Approaches for Adolescent Substance Use Disorders. Child Adol Psych Cl. 2010;19(3):547-+.

24. Letourneau EJ, McCart MR, Sheidow AJ, Mauro PM. First Evaluation of a Contingency Management Intervention Addressing Adolescent Substance Use and Sexual Risk Behaviors: Risk Reduction Therapy for Adolescents. J Subst Abuse Treat. 2017;72:56-65.

25. Mitchell JT, LeGrand S, Hightow-Weidman LB, et al. Smartphone-Based Contingency Management Intervention to Improve Pre-Exposure Prophylaxis Adherence: Pilot Trial. Jmir Mhealth and Uhealth. 2018;6(9).

26. Erguera XA, Johnson MO, Neilands TB, et al. WYZ: a pilot study protocol for designing and developing a mobile health application for engagement in HIV care and medication adherence in youth and young adults living with HIV. BMJ open. 2019;9(5).

27. Saberi P, McCuistian C, Agnew E, et al. Video-Counseling Intervention to Address HIV Care Engagement, Mental Health, and Substance Use Challenges: A Pilot Randomized Clinical Trial for Youth and Young Adults Living with HIV. Telemed Rep. 2021;2(1):14-25.

28. Saberi P, Berrean B, Milionis C, Wong JO, Arnold EA. We are family: designing and developing a mobile health application for the San Francisco bay area House Ball and Gay Family communities. mHealth. 2020.

29. Saberi P, Lisha NE, Erguera XA, et al. A Mobile Health App (WYZ) for Engagement in Care and Antiretroviral Therapy Adherence Among Youth and Young Adults Living With HIV: Single-Arm Pilot Intervention Study. JMIR Form Res. 2021;5(8):e26861.

30. Saberi P, Ming K, Legnitto D, Neilands TB, Gandhi M, Johnson MO. Feasibility and acceptability of novel methods to estimate antiretroviral adherence: A longitudinal study. Plos One. 2019;14(1).

31. Kann L, McManus T, Harris WA, et al. Youth Risk Behavior Surveillance - United States, 2017. MMWR Surveill Summ. 2018;67(8):1-114.

32. Wang Y, Sloan FA. Present bias and health. J Risk Uncertain. 2018;57(2):177-198.

33. Jairath N, Hogerney M, Parsons C. The role of the pilot study: a case illustration from cardiac nursing research. Applied nursing research : ANR. 2000;13(2):92-96.

34. Lancaster GA, Dodd S, Williamson PR. Design and analysis of pilot studies: recommendations for good practice. Journal of evaluation in clinical practice. 2004;10(2):307-312.

35. Kraemer HC, Mintz J, Noda A, Tinklenberg J, Yesavage JA. Caution regarding the use of pilot studies to guide power calculations for study proposals. Arch Gen Psychiat. 2006;63(5):484-489.

36. Becker PT, Schulte HD. Publishing pilot intervention studies. Res Nurs Health. 2008;31(1):1-3.

37. Kistin C, Silverstein M. Pilot Studies A Critical but Potentially Misused Component of Interventional Research. Jama-J Am Med Assoc. 2015;314(15):1561-1562.

38. Creswell JW, Creswell JD. Research design : qualitative, quantitative, and mixed methods approaches. Fifth edition. ed. Los Angeles: SAGE; 2018.
